# British and American Hospitals

**Published:** 1906-07-14

**Authors:** William Epps

**Affiliations:** Secretary Royal Prince Alfred Hospital, Sydney.


### BRITISH AND AMERICAN HOSPITALS.

FROM AN AUSTRALIAN STANDPOINT. By William Epps, Secretary Royal Prince Alfred Hospital, Sydney.

I think it will be admitted that comparison is the best test of value. A man may observe a thing day after day, year after year, and be satisfied that it is the best to be obtained. Then, by chance perhaps, he will see something similar, but better, somewhere else, and he has satisfied himself that his best was after all but a second or third best. In other words, he has obtained a new standard. That is the value of comparison. When little less than 12 months ago, by the direction of the Board of Directors of the Royal Prince Alfred Hospital, the writer set out to investigate the methods of management of hospitals in the English-speaking communities of America and Europe, he had but the Australian standard, with which he had been well satisfied. Now he has a different standard, and sees things which previously seemed all right with a more seeing eye. If there is one lesson to be learned by his visit, from which others may benefit, it is the value of seeing what others are doing—not necessarily to copy them, often, indeed, to avoid this.

This lesson has been learned in America. There hospital managers are constantly interchanging visits and comparing notes, while annually they meet together in conference and try to suck each other's brains. In England the writer found that in many cases the head of one great hospital had not even been inside another great hospital—practically just round the corner. In Australia the population is so scattered, and distances are so great, that the possibilities of personal interchanges of ideas are few; and in any case the large hospitals are small in numbers. Certainly, both in England and Australia, some organisation on the lines of the Association of Hospital Superintendents of the U.S.A. and Canada would add greatly to the sum of knowledge of those who have to do with the administration of these institutions, and probably raise the standard all round.

What is the great point impressed upon the observer after a tour of the large hospitals of the English-speaking world? Without a doubt it is the fact that, though in one essential all are travelling on one main line—namely, that of utilising the services of the leading medical and surgical experts for the cure of the sick poor upon an honorary basis—the method of providing the means of maintenance in England, America, and Australia is quite different. In England, with one or two notable exceptions, and then only in one particular, patients are treated free. In America a proportion of the patients are "free" patients, but a very large proportion of the revenue is derived from patients themselves. In Australia the State enters largely into the finances of all the large hospitals. Approximately, the Canadian institutions, as a rule, work along United States lines, but with a tendency to Australian methods in some particulars. In each of these comparisons general hospitals alone are treated of.

It certainly was a surprise to the writer to find that so much consideration was given in America to the private or paying patient. As to how far this has been a modern or recent development, it did not occur to him to inquire. His interest at the time was rather with methods than ethics, and the generalisation made above is rather the result of subsequent reflection and comparison of figures than of immediate impression. One thing, however, seems to impress itself upon the visitor as he passes through—the fact that in all the hospitals outside the few provided for by the municipalities, such as the Cook County at Chicago, the Bellevue in New York, the Boston City at Boston, and the Philadelphia at Philadelphia, the paying patient seems to be the pivot on which all finance turns. If he is prepared to come forward in sufficient numbers, and to pay the prices demanded, the hospital can get along all right, and has not to go to the public for support; otherwise there is a constant struggle for existence. In the case of the four hospitals I have enumerated these conditions do not apply. They are supported from the rates, and are absolutely free. Likewise they are the great hospitals of America, from the point of view of size, as each has about 1,000 beds or over. It may be also that, from the medical standpoint, they are in the forefront. That aspect of the matter did not concern me, as a layman looking for "wrinkles" in management and administration. Certainly, with one marked exception, their general methods and equipment did not appear to compare specially favourably with those of what may be termed the voluntary institutions, and they are not quoted in America as examples of how to do things. I shall not refer to them further in this connection than to say that, with the exception of the Boston City, which is a brilliant exception to the rule, these municipal institutions appear to be huge uninteresting places, something similar to the English workhouse infirmaries, but no doubt doing a good medical work.

To return to the general hospitals proper, such as we understand them, it would appear that originally their founders generally had before them the idea of a free charity, as endowments on a fairly lavish scale have been provided. These bring in generally fair incomes, which form the foundation of the means of carrying on the hospitals. These endowments, with the hospital buildings and equipment, constitute what may be termed the capital and stock in trade of the institutions, and with this nucleus the managers set out to provide, on a fairly business basis, for the sick of their towns. I purposely do not say the *sick poor*, as we speak of them in England and Australia. The sick of all classes are here taken into consideration, and at this point the American and (to a lesser extent) the Canadian hospital managers diverge from their confrères in Great Britain and Australia. So far as I could gather, those responsible for the management of the institutions go into Committee of Ways and Means each year, having in view the actual revenue in sight from endowments and the number of beds. Should the income be sufficient to keep open all the beds for the sick poor free of payment, well and good. There is no need to go outside for further assistance, though, as in Great Britain, these hospitals are very few and far between. If the income in sight is not sufficient, hospital directors do not, as we do, go out into the highways and by-ways, establish Hospital Saturday and Sunday Funds, King Edward Funds, and so on, and drag money out of the charitable rich. In a few cases something of this kind is done, notably in the Presbyterian hospitals in Chicago and New York, and the St. Luke's and Mount Sinai in New York, which are more or less sectarian institutions in their inception, though not in the matter of their patients, and receive considerable support from the members of their respective denominations.

But even here again the collections from the charitable public form but a comparatively small proportion of the whole income. Instead of closing up that proportion of the beds which they cannot afford to maintain, the hospital directors set aside a proportion of them for the use of those who can afford to pay for treatment, and charge fees sufficient to enable them to maintain the complete hospital without recourse to the charitable public. In this way provision is made within the walls of one institution for all classes of the community. Of course, the methods of admission and payment vary. In some cases the patients are divided by sharp lines into private and free, with a severe discrimination between the two classes, both as to accommodation and diet. In others there is a more gradual scheme of demarcation, and the dividing lines are "private," "semi-private," and "free." In others, again, there are what are termed "paying" patients coming in between "semi-private" and "free," or taking the place of the former. These "paying patients" generally contribute a fixed sum of, say, one dollar per diem. Indeed, in some hospitals there are but the two classes of "paying" and "free." However, the main point is that in one form or another nearly all the hospitals of Canada and the United States, outside the municipal and fully endowed institutions, have two classes of patients.

(*To be continued.*)

